# Marked Mild Cognitive Deficits in Humanized Mouse Model of Alzheimer’s-Type Tau Pathology

**DOI:** 10.3389/fnbeh.2021.634157

**Published:** 2021-05-21

**Authors:** Joshua D. Cho, Yoon A. Kim, Elizabeth E. Rafikian, Mu Yang, Ismael Santa-Maria

**Affiliations:** ^1^Taub Institute for Research on Alzheimer’s Disease and the Aging Brain, Columbia University, New York, NY, United States; ^2^Department of Pathology & Cell Biology, Columbia University, New York, NY, United States; ^3^The Mouse NeuroBehavior Core, Institute for Genomic Medicine, Columbia University, New York, NY, United States

**Keywords:** cognition, spatial memory, tau pathology, mouse model, Alzheimer’s disease, neurodegeneration

## Abstract

Hyperphosphorylation and the subsequent aggregation of tau protein into neurofibrillary tangles (NFTs) are well-established neuropathological hallmarks of Alzheimer’s disease (AD) and associated tauopathies. To further examine the impact and progression of human tau pathology in neurodegenerative contexts, the humanized tau (htau) mouse model was originally created. Despite AD-like tau pathological features recapitulated in the htau mouse model, robustness of behavioral phenotypes has not been fully established. With the ultimate goal of evaluating the htau mouse model as a candidate for testing AD therapeutics, we set out to verify, in-house, the presence of robust, replicable cognitive deficits in the htau mice. The present study shows behavioral data collected from a carefully curated battery of learning and memory tests. Here we report a significant short-term spatial memory deficit in aged htau mice, representing a novel finding in this model. However, we did not find salient impairments in long-term learning and memory previously reported in this mouse model. Here, we attempted to understand the discrepancies in the literature by highlighting the necessity of scrutinizing key procedural differences across studies. Reported cognitive deficits in the htau model may depend on task difficulty and other procedural details. While the htau mouse remains a unique and valuable animal model for replicating late onset AD-like human tau pathology, its cognitive deficits are modest under standard testing conditions. The overarching message is that before using any AD mouse model to evaluate treatment efficacies, it is imperative to first characterize and verify the presence of behavioral deficits in-house.

## Introduction

Alzheimer’s disease (AD) is a chronic neurodegenerative disease characterized by cognitive impairment, progressive memory loss, dementia, and behavioral disturbances (Jack et al., 2018). On a molecular level, extracellular deposits of amyloid beta and the resulting neuritic plaques along with the intracellular accumulation of hyperphosphorylated tau protein into paired helical filaments (PHFs) and neurofibrillary tangles (NFTs) contribute to neuronal loss and the pathological hallmarks seen in AD brains (De Strooper and Karran, 2016). The severity of tau pathology, in particular, has been implicated as a major predictive factor for the extent of cognitive impairment, making it a promising therapeutic target for AD (Arriagada et al., 1992; Bejanin et al., 2017; Harrison et al., 2019; La Joie et al., 2020).

As one of the most abundant microtubule-associated proteins in the central nervous system, tau has been recognized to play major roles in promoting microtubule assembly and stabilization and in maintaining the normal morphology of neurons. Recent studies suggest that tau, upon alternative mRNA splicing, may also participate in the regulation of intracellular signaling pathways, development, and the viability of neurons (Avila et al., 2004; Wang and Mandelkow, 2016). In the human CNS, tau protein is translated from a 6-kb mRNA transcript generating a series of six tau protein isoforms of 37–46 kDa which result from alternative splicing of exons 2, 3, and 10 (Guo et al., 2017). The complexity of the tau isoforms is further increased by various posttranslational modifications (Wang and Liu, 2008). Thus, dysregulation of tau proteostasis can arise from a number of sources such as: splice site and/or missense mutations, changes in overall expression or isoform composition (Trabzuni et al., 2012; Strang et al., 2019), various post-translational modifications (Mair et al., 2016; Arakhamia et al., 2020), and epigenetic regulators (Lardenoije et al., 2015; Millan, 2017). These changes can lead to the abnormal aggregation of tau, resulting in the eventual seeding and spread of pathological tau species that contribute to a number of neurodegenerative diseases, collectively known as tauopathies, which includes AD (Gotz et al., 2019).

Therefore, elucidating the specific role of tau protein is imperative to unraveling the underlying mechanisms of these neurodegenerative diseases. To address this issue, the laboratory of Peter Davies created the htau mouse model (Andorfer et al., 2003). The htau mouse produces all six isoforms of human tau with no evidence of native, murine tau expression. Furthermore, htau mice develop pathologies that effectively model spatiotemporal and histopathological features seen in AD, such as age-dependent hyperphosphorylation of tau in the hippocampus and frontal cortex, somatic redistribution of tau to dendritic compartments, tau aggregation into NFT-like structures, increased inflammation, decreased cortical thickness, and neuronal cell death (Andorfer et al., 2003). However, although the htau mouse model elegantly recapitulates many of the histopathological features of AD, behavioral analyses of this model have yielded inconsistent results in the past (Table 1). Taking Morris Water Maze, one of the most commonly used tests in studies of AD models, as an example, half of the published studies reported cognitive deficits in htau mice, whereas the other half reported normal learning/memory (Supplementary Table 1). This lack of consistency led us to conduct the current study and verify the presence/robustness of cognitive phenotypes in this mouse model before using it to screen for novel treatment strategies. While mouse models have provided new insights into disease mechanisms, efforts to convert this knowledge into gains in the clinic have struggled. One of the main sources of concern is the animal models used in preclinical drug development. Thus, we would like to emphasize here that the value of our study goes beyond serving as a pivotal point of phenotypic characterization by providing the research community with key insights into critical factors that might substantially affect replicability of “disease phenotypes” and subsequent preclinical research on treatment development. Also, we attempted to understand our results in the context of existing literature, and to provide some insights on reproducibility of behavioral data.

**TABLE 1:** Chronological behavior analysis of the htau mouse model.

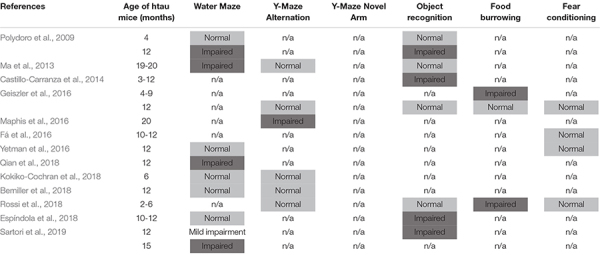

*Summary of studies reporting behavioral measures on the htau mouse model. Normal Cognitive Performance indicated by Light Grey Shading. Impaired Cognitive Performance indicated by Dark Grey Shading.*

## Materials and Methods

### Animals

Male and female control mice (C57BL/6J; Stock #000664) and htau mice (Polydoro et al., 2009) (B6.Cg-*Mapt^TM 1(EGFP)Klt^* Tg(MAPT)8cPdav/J; Stock #005491) were obtained from The Jackson Laboratory (Bar Harbor, Maine). All mice were group-housed by sex (3–5 per cage, standard polypropylene cages placed on ventilated rack) in a temperature and humidity-controlled vivarium at Columbia University Medical Center and maintained on a standard 12 h light/dark cycle (lights on at 7 am) with food and water provided *ad libitum*. A piece of Nestlets was placed in each cage as enrichment. Htau mice were backcrossed 10 times to the C57BL/6J background. Behavioral testing was performed in htau mice and age-matched C57BL/6J mice (non-littermates from JAX). All behavioral experiments were performed by a male experimenter during the light phase (between 10 am and 4 pm) in accordance with national guidelines (National Institutes of Health) and approved by the Institutional Animal Care and Use Committee of Columbia University.

### Immunohistochemistry

Detailed protocol can be found in Supplementary Methods.

### Behavioral Tests

Behavioral testing was performed following previously published and established protocols (Yang et al., 2012; Shore et al., 2020). Detailed protocols can be found in Supplementary Methods. In general, tests progressed from the least stressful (open field) to the most stressful (water maze, fear conditioning). A Two-way RM ANOVA, with time and genotype as the two factors, was performed on distance traveled, center time, and vertical movement data collected from the Open Field test. For short-term novelty preference in Y-Maze, an unpaired *t*-test or non-parametric Mann–Whitney U test was carried out to analyze preference index (time spent in novel arm/time spent in novel arm + time spent in familiar arm). In the Morris Water Maze, latency to platform, swim distance, and swim speed were all analyzed using Two-way RM ANOVA with Day as the within-subject factor and Genotype as the between subject factor. Mean %time spent in the trained quadrant vs. other qudrants during probe trials 1 (2 h after the last training trial) and 2 (24 h after the last training trial) in the Morris Water maze were analyzed using paired t test, to detect preference. Platform crossings during the probe trials were analyzed using an Unpaired *t*-test. Percentage of time spent freezing during the contextual and cued phases of Fear Conditioning was analyzed using an unpaired *t*-test.

## Results

No sex differences were found in any test employed in the current study. See Supplementary Material for statistical results of sex comparisons.

### Impaired Short-Term Spatial Novelty Preference in Old htau Mice

**FIGURE 1:**
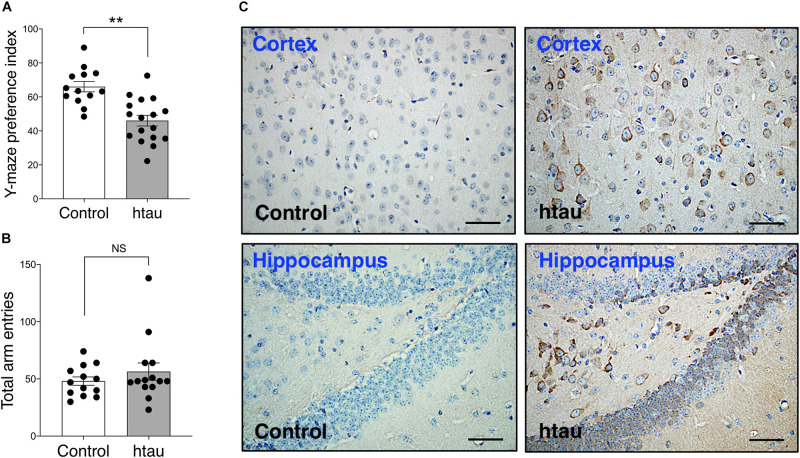
Htau mice show impaired spatial novelty preference in Y Maze. **(A)** Preference index was significantly reduced in 16-18 months htau mice in comparison to controls. **(B)** Total arm entries are similar in htau and controls during the Y maze test, ruling out motor confounds. **p < 0.01 vs. control (control, *N* = 13; htau, *N* = 17). **(C)** Representative immunohistochemistry images from the cortex and hippocampus showing accumulation of tau pathology (stained with phospho-specific tau AT8 antibody) in old htau mice compared with age matched control mice. Brain areas shown are involved in the task performance. Scale bars: 120 μm. All values are expressed as means ± SEM.

As shown in Figure 1A, 16–18 months old htau mice exhibited impaired spatial novelty preference in the Y maze test. An unpaired *t*-test revealed a significantly impaired preference index in htau mice as compared to controls (*t* = 4.47, *p* = 0.0001). Total arm entries were not significantly different between genotypes (Figure 1B, Mann–Whitney U Statistic = 77.500, NS). No sex differences were found in either control (*t* = 0.59, NS) or htau (*t* = 0.19, NS), hence sexes were combined in data illustrated in Figure 1A. Additionally, we confirmed that pathological tau alterations in cortical and hippocampal areas were present in the brains of htau mice; observations that agree with previously published analyses of this mouse (Andorfer et al., 2003), resembling neuropathological features observed in late onset AD brains (Hyman et al., 2012; Montine et al., 2012; DeTure and Dickson, 2019; Figure 1C).

### Mostly Normal Morris Water Maze Performance in Old htau Mice

**FIGURE 2:**
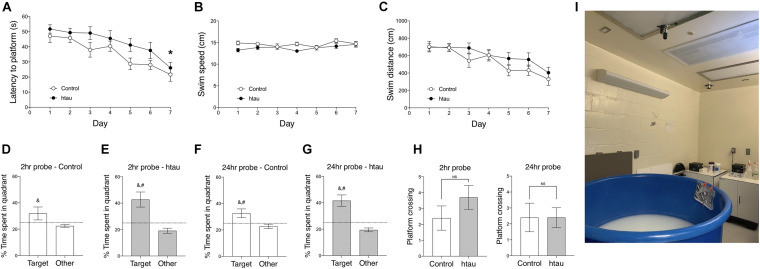
Htau mice exhibited normal learning and memory in the Morris Water Maze. **(A)** Latency to platform was significantly longer in 20-month-old htau than in control mice across training days which may be related to marginally slower swim speed in the htau mice **(B)**. No genotype differences were found in swim distance **(C)**. *p < 0.05 vs. controls (control, *N* = 10; htau, *N* = 10). **(D–G)** In probe trial 1, performed two hours after the last training trial, and in probe trial 2 performed 24 h later, htau mice exhibited a significant preference for the trained quadrant, showing a higher %time in the Target quadrant than in other quadrants and as compared to the chance level. Control mice exhibited similar preference, except for the insignificant difference between Target and chance in probe trial 1. ^&^*p* < 0.05 vs. Other; ^#^*p* < 0.05 vs. chance **(H)** No significant differences were found in number of platform crossings. **(I)** proximal and distal cues of the water maze. All values are expressed as means ± SEM.

As shown in Figure 2, in the Morris Water Maze test, latency to reach the hidden platform was significantly longer in 20-month-old htau mice than in age-matched controls (Figure 2A) (Two-way RM ANOVA, main effect of genotype difference across days: *F*_1,18_ = 8.42, *p* < 0.01). Note that this difference in latency might be related to a close-to-significant trend of htau mice swim slower than control mice (Figure 2B, *F*_1,18_ = 3.85, *p* = 0.065, NS) and may not reflect learning deficit *per se*. As described below, htau mice exhibited a trend of having higher locomotor activity in the open field test, and we speculate that the slower speed is unlikely to be the result of gross motor defects. Distance swam was similar in both genotypes (Figure 2C, *F*_1,18_ = 2.78, NS). To see if the animals progressed significantly across days as a result of training, we analyzed the latency data in each genotype using one-way RM ANOVA. The main effect of “Day” was significant in controls (*F*_6,54_ = 6.80, *p* < 0.001) as well as in htau mice (*F*_6,54_ = 4.75, *p* < 0.001), indicating improved searching as the result of training in both groups (Figure 2A). Percentage of time spent in the trained quadrants and other quadrants during probe trials were analyzed using paired t test within each genotype, to detect preference for the trained quadrant.

In probe trial 1 (Figures 2D,E) control mice spent significantly more time in the target quadrants than in other quadrants (*t* = 2.03,*p* < 0.049) but the difference between %time in the target quadrant and chance level was not significant (*t* = 1.41, *p* = 0.18, NS). htau mice did spend more time in the target quadrant over other quadrants (*t* = 4.46, *p* < 0.001) and the difference between %time in the target quadrant and chance level was significant (*t* = 3.11, *p* < 0.001). In probe trial 2 (Figures 2F,G), both controls and htau mice spent more time in the target quadrant over other quadrants (control: *p* < 0.01; htau: *p* < 0.001) and the difference between %time in the target quadrant and chance level was significant for both groups (control: *p* < 0.05; htau: *p* < 0.001).

These data indicate that both genotypes preferred the quadrant in which the hidden platform used to be located in. The number of platform crossings was not different between genotypes in probe trial 1 (*t* = 1.22, NS, Figure 2H) or probe trial 2 (*t* = 0.00, NS, data not shown). These data indicate normal spatial learning memory in 20 months old htau mice tested in the widely used, standard water maze protocol.

### Normal Contextual and Cued Fear Conditioning in Old htau Mice

**FIGURE 3:**
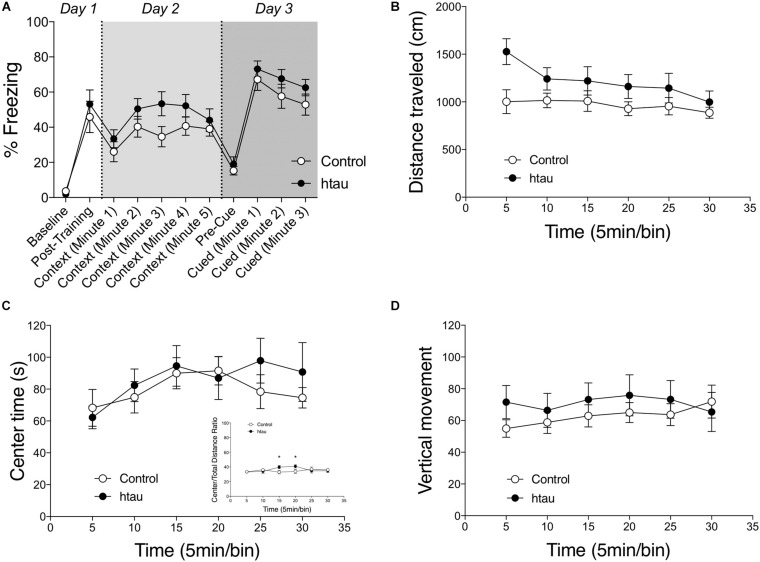
Normal fear conditioning, anxiety-like behaviors and spontaneous locomotor activity in htau mice. **(A)** 16–18 months old htau mice exhibited a normal freezing response in the fear conditioning test. No genotype differences were found in baseline freezing, post-shock freezing, contextual freezing, pre-cue freezing, or cued freezing (control, *N* = 12; htau, *N* = 14). **(B)** A trend was observed for htau mice to travel a longer distance in a 30-minutes open field test. **(C)** No genotype differences were found in time spent in the center zone of the arena. The ratio of center/total distance was transiently higher in htau mice (C insert). These data indicated normal anxiety-like behaviors in htau. **(D)** No genotype differences were found in number of vertical movements (control, *N* = 10; htau, *N* = 10). All values are expressed as means ± SEM.

We next examined contextual and cued fear conditioning. As shown in Figure 3A, 16–18 months old htau mice exhibited normal %freezing in the contextual conditioning test. Two-way RM ANOVA revealed a mild but insignificant trend for htau mice to exhibit a higher %freezing in the contextual conditioning test (*F*_1,24_ = 3.78, *p* = 0.078, NS). The main effect of time (five 1 min bins) was significant (*F*_4,24_ = 4.00, *p* < 0.01) but the interaction of time × genotype was not significant (*F*_4,24_ = 0.60, NS). In the cued conditioning test, two-way RM ANOVA did not reveal a genotype effect for %freezing (*F*_1,24_ = 1.60, NS). The main effect of time (min) was significant (*F*_4,24_ = 6.24, *p* < 0.01), but the interaction of time x genotype was not significant (*F*_4,24_ = 0.20, NS). Pre-shock freezing is close to zero for both genotypes, and the difference was not significant (Mann–Whitney = 48.00, NS). Post-shock freezing did not differ between genotypes (Non-parametric Mann–Whitney U = 67.00, NS), indicating comparable sensitivity to foot shocks and/or reactivity to the fear stimulus. In both genotypes, two-way RM ANOVA indicated that cued freezing is significantly higher than pre-cue freezing (control, *F*_1,11_ = 61.72, *p* < 0.001; htau, *F*_1,13_ = 87.78, *p* < 0.001).

### Normal Spontaneous Exploratory Activity

As shown in Figures 3B–D, 16–18 months old htau mice exhibited normal open field locomotor activity. Two-way RM ANOVA did not reveal a significant main effect of genotype on distance traveled (*F*_1,18_ = 3.11, *p* = 0.095, with htau mice showing a trend towards increased activity). Vertical movements (*F*_1,18_ = 0.43, NS) and time spent in the center zone (*F*_1,18_ = 0.32, NS) were also comparable between controls and htau mice. These data ruled out any gross motor abnormalities as confounding factors in the aforementioned learning and memory tests. The ratio of distance moved in the center arena vs. total distance moved was calculated for the first 10 min and the entire 30 min test session. The main effect of genotype was not significant in the first 10 min (two-way RM ANOVA, *F*_1,18_ = 0.266, NS). Although the main effect of genotype was not significant over the 30-min session either (*F*_1,18_ = 0.357, NS), a significant genotype × time bin interaction was found (*F*_1,18_ = 2.867, *p* = 0.019). *Post hoc* analysis indicated that the ratio was transiently higher in control at the 3rd and (*p* < 0.05) and 4th (*p* < 0.05) time bin. These results are illustrated in Figure 3C.

## Discussion

Cognitive phenotypes in htau mice have not been consistently reported in the past decade (Table 1). Overall, the existing literature suggests that the cognitive phenotype in aged htau mice, with standard behavioral testing methods, is not robust. Consequently, in the present study we set out to evaluate learning and memory in our own colony of aged htau mice. We tested 16–20 months old htau mice and age-matched controls in a battery of learning and memory tests commonly used to assess AD-like phenotypes in mouse models. While it is interesting that we found an impaired spatial recognition memory in aged htau mice tested in the Y maze, our study aslo revealed normal Morris Water Maze and fear conditioning performances, adding to the body of existing evidence that failed to show robust cognitive impairments in htau mice (Table 1). It is notable that in Morris Water Maze, our control mice failed to display a clear preference for the trained quadrant in probe trial 1 (Figure 2). This could be partially attributable to stress and fatigue from the training trials earlier in the day. Although both groups exhibited preference for the trained quadrant in probe trial 2, it is possible that the probe trial performed 2 h after the last training trial could have diminished the strength of preference exhibited in the probe trial performed 24 h later. This is due to the extinction effect, in the absence of a “refresher trial” between the two probe tests.

Given that the water maze is usually considered one of the most sensitive tests for detecting functional defects of the hippocampus (Vorhees and Williams, 2006; Kraeuter et al., 2019), we compared important parameters employed in each experiment, in an effort to comprehend why only about half of previous studies reported water maze deficits in htau mice. We noticed that the ratio of pool-to-platform size is 160 in studies that reported deficits, but only 110 in studies that reported no deficits. Interestingly, age or sex does not seem to be factors that affect result outcome (Supplementary Table 1). We argue that the larger the ratio of pool-to-platform size, the more difficult the task is. As such, the discrepancies in previous findings may be partially explained by task difficulty (which is related to pool-to-platform ratio).

Y maze task difficulty is higher than the water maze, due to its lack of strong incentive stimuli and repetitions. In the Y-Maze novel arm preference test, there is only one non-rewarded “learning trial” before the memory test is performed. In contrast, Morris Water Maze utilizes a strong incentive (escaping the water) and allows the animal multiple days of learning (with four trials/day). These important differences between Y-maze and Morris Water Maze may be helpful to understand why we discovered a deficit in the Y-maze test but not in the Morris Water Maze test. The fear conditioning test also utilizes a salient aversive stimulus—three 2-s foot shocks—during the conditioning phase. Therefore, behavioral tests that incorporate strong aversive stimuli might not be particularly sensitive to subtle differences in learning and memory performance (Redish and Touretzky, 1998; Broadbent et al., 2006).

Given the literature and our own data, we reason that cognitive impairments in htau mice may be too subtle for standard testing procedures to detect. Instead of arguing which tests may be more or less sensitive, we suggest that incorporating a number of different memory tests is a good strategy to ensure that multiple facets of the cognitive phenotype are comprehensively evaluated. In addition, resource permitting, it could be useful to incorporate low-stress operant tests such as the Bussey–Saksida touchscreen system (Horner et al., 2013; Mar et al., 2013; Oomen et al., 2013) which has high technical and conceptual similarities with the CANTAB Alzheimer’s test battery used to assess cognitive function in human AD patients (Swainson et al., 2001; Egerhazi et al., 2007).

Lastly, we want to emphasize the translational value of our novel findings. In this regard, using this humanized mouse model of late onset Alzheimer’s tau pathology, we found a spatial recognition memory impairment that might be relevant to some of the key cognitive deficits observed in patients with AD and related dementias. Spatial navigation, in particular, is emerging as a potential cost-effective biomarker to detect cognitive decline in incipient AD and related dementias in the preclinical stages, which has important implications for future diagnostics and treatment approaches (Coughlan et al., 2018).

## Data Availability Statement

The raw data supporting the conclusions of this article will be made available by the authors, without undue reservation.

## Ethics Statement

The animal study was reviewed and approved by Institutional Animal Care and Use Committee of Columbia University.

## Author Contributions

IS-M and MY supervised the project. JC, YK, and ER performed research and conducted behavioral experiments. JC, MY, and IS-M analyzed and interpreted data and wrote the manuscript. All authors contributed to the article and approved the submitted version.

## Conflict of Interest

The authors declare that the research was conducted in the absence of any commercial or financial relationships that could be construed as a potential conflict of interest.
