# Supplementary material for: Marked Mild Cognitive Deficits in Humanized Mouse Model of Alzheimer’s-Type Tau Pathology
Source: Front Behav Neurosci. 2021 May 21;15:634157. doi: 10.3389/fnbeh.2021.634157 (PMC8175658; doi:10.3389/fnbeh.2021.634157)
Supplement: Supplementary file 1 [file Data_Sheet_1.pdf]

Supplementary Information

Marked mild cognitive deficits in humanized mouse model of Alzheimer's-type tau pathology

Joshua D. Cho[1,2], Yoon A. Kim[1,2,#], Elizabeth E. Rafikian[3,#], Mu Yang[3], Ismael Santa-Maria[1, 2,] *

Affiliations:
[1] Taub Institute for Research on Alzheimer's Disease and the Aging Brain, Columbia University, New York, NY, USA.
[2] Department of Pathology & Cell Biology, Columbia University, New York, NY, USA.
[3] The Mouse NeuroBehavior Core. Institute for Genomic Medicine, Columbia University, New York, NY, USA

[#] These authors contributed equally to this work.

* Correspondence:

Ismael Santa-Maria, Department of Pathology and Cell Biology, Columbia University, 630 West 168th Street, PS15-408, New York, NY, 10032, is2395@cumc.columbia.edu

Supplementary tables

Supplementary Table 1. Studies of Morris Water Maze Performance in htau Mice.

Author (first)	*Year*	*Age (months)*	*Sexes Tested*	*Training Regimen*	*Inter-trial Interval*	*Probe*	*Water Maze Performance*	*Pool Diameter*	*Platform Size*	*Pool/Platform Ratio*
Polydoro M., et al.	2009	12	M	3 trials/day for 6 days	20 min.	6 hrs. after last trial	Impaired	110	78.5	121
Ma Q-L., et al.	2013	19-20	M & F	4 trials/day for 5 days	15 sec.	24 hrs. after last trial	Impaired	152	176.6	103
Qian S., et al.	2018	12	Not specified	3 trials/day for 4 days	20 sec.	24 hrs. after last trial	Impaired	180	132.7	192
Sartori M., et al.	2019	15	M & F	4 trials/day for 6 days	90 min.	24 hrs. after last trial	Impaired	150	78.5	225
Average		14.63								160.25
Yetman M.J., et al.	2016	12	M & F	4 trials/day for 5 days	15 min.	Every day after last trial	Normal	122	100	117
Bemiller S.M., et al.	2018	12	Not specified	4 trials/day for 5 days	n/a	No probe	Normal	122	100	117
Espindola S.L., et al.	2018	12	M		10 min.	24 hrs. after last trial	Normal	100	n/a	n/a
Cho J.D., et al.	2020	20	M & F	4 trials/day for 5 days	1 min.	2 & 24 hrs. after last trial	Normal	122	133	88
Average		14.00								107.3

Supplementary Table 2. Statistical Tests for Sex Differences within Genotype

Behavioral Test	*Genotype*	*Statistical Test*	*F/t*	*P value*
Open Field (Distance)	Control	Two-way RM ANOVA (Time x Sex)	$F_{5,40}$ = 8.18	<0.0001
	Control	Two-way RM ANOVA (Sex)	$F_{1,8}$ = 1.50	0.256
	htau	Two-way RM ANOVA (Time x Sex)	$F_{5,40}$ = 1.60	0.183
	htau	Two-way RM ANOVA (Sex)	$F_{1,8}$ = 0.26	0.626
Open Field (Center Time)	Control	Two-way RM ANOVA (Time x Sex)	$F_{5,40}$ = 1.26	0.299
	Control	Two-way RM ANOVA (Sex)	$F_{1,8}$ = 0.35	0.571
	htau	Two-way RM ANOVA (Time x Sex)	$F_{5,40}$ = 0.35	0.878
	htau	Two-way RM ANOVA (Sex)	$F_{1,8}$ = 0.32	0.586
Open Field (Vertical Movement)	Control	Two-way RM ANOVA (Time x Sex)	$F_{5,40}$ = 0.80	0.554
	Control	Two-way RM ANOVA (Sex)	$F_{1,8}$ = 0.13	0.731
	htau	Two-way RM ANOVA (Time x Sex)	$F_{5,40}$ = 2.68	0.035
	htau	Two-way RM ANOVA (Sex)	$F_{1,8}$ = 4.19	0.075
Open Field (Center:Total Distance Ratio)	Control	Two-way RM ANOVA (Time x Sex)	$F_{5,40}$ = 2.08	0.087
	Control	Two-way RM ANOVA (Sex)	$F_{1,8}$ = 0.04	0.854
	htau	Two-way RM ANOVA (Time x Sex)	$F_{5,40}$ = 0.34	0.885
	htau	Two-way RM ANOVA (Sex)	$F_{1,8}$ = 0.45	0.522
Novel Arm Y-Maze	Control	Unpaired t test	t = 0.59	0.066
	htau	Unpaired t test	t = 0.19	0.309
Fear Conditioning (All stages)	Control	Two-way RM ANOVA (Time x Sex)	$F_{12,120}$ = 1.00	0.454
	Control	Two-way RM ANOVA (Sex)	$F_{1,10}$ = 1.08	0.324
	htau	Two-way RM ANOVA (Time x Sex)	$F_{12,144}$ = 1.33	0.231
	htau	Two-way RM ANOVA (Sex)	$F_{1,12}$ = 1.89	0.193
MWM (Latency)	Control	Two-way RM ANOVA (Time x Sex)	$F_{6,48}$ = 1.43	0.223
	Control	Two-way RM ANOVA (Sex)	$F_{1,8}$ = 2.83	0.131
	htau	Two-way RM ANOVA (Time x Sex)	$F_{6,48}$ = 1.31	0.273
	htau	Two-way RM ANOVA (Sex)	$F_{1,8}$ = 0.79	0.401
MWM (Speed)	Control	Two-way RM ANOVA (Time x Sex)	$F_{6,48}$ = 0.83	0.550
	Control	Two-way RM ANOVA (Sex)	$F_{1,8}$ = 0.27	0.619
	htau	Two-way RM ANOVA (Time x Sex)	$F_{6,48}$ = 2.01	0.083
	htau	Two-way RM ANOVA (Sex)	$F_{1,8}$ = 3.63	0.093
MWM (Distance)	Control	Two-way RM ANOVA (Time x Sex)	$F_{6,48}$ = 0.79	0.580
	Control	Two-way RM ANOVA (Sex)	$F_{1,8}$ = 3.98	0.081
	htau	Two-way RM ANOVA (Time x Sex)	$F_{6,48}$ = 1.59	0.170
	htau	Two-way RM ANOVA (Sex)	$F_{1,8}$ = 1.84	0.212
MWM (2hr Probe)	Control	Two-way RM ANOVA (Time x Sex)	$F_{2,16}$ = 1.82	0.194
	Control	Two-way RM ANOVA (Sex)	$F_{1,8}$ = 1.82	0.215

	htau	Two-way RM ANOVA (Time x Sex)	$F_{2,16} = 0.76$	0.482
	htau	Two-way RM ANOVA (Sex)	$F_{1,8} = 0.76$	0.407
MWM (24hr Probe)	Control	Two-way RM ANOVA (Time x Sex)	$F_{2,16} = 0.06$	0.941
	Control	Two-way RM ANOVA (Sex)	$F_{1,8} = 0.06$	0.811
	htau	Two-way RM ANOVA (Time x Sex)	$F_{2,16} = 0.19$	0.830
	htau	Two-way RM ANOVA (Sex)	$F_{1,8} = 0.19$	0.676

Supplementary methods

Subjects used in behavioral tests were between 16 and 20 months of age. In cases when the same animals were tested in multiple tests, the order of tests was: Open field →Y maze →Fear conditioning →Morris Water Maze, with 1-week intervals between open field and Y maze and between Y maze and Fear conditioning. After Fear conditioning, animals were home caged for a least 1 month before the Morris Water Maze task was performed.

Y-Maze Novel Arm Preference Test

The Y-Maze is a standard behavioral test for assessing short term spatial reference memory based on the rodent's natural tendency to explore novel locations (Sukoff Rizzo et al., 2018). Memory impairment is indicated by failing to spend more time exploring the novel arm than the familiar arm. The test was conducted in the Y maze apparatus (Maze Engineer, Skokie, IL), which consists of three arms of equal length (35 cm), arm lane width (5 cm), and wall height (10 cm). A 2 cm x 2 cm sticker (an equal sign, a bus, and a plane) is taped at the end of each lane, one inch above the floor. The start arm is always marked with the equal sign, and the bus and the plane stickers are counter balanced in the familiar and the novel arm. In Trial 1, each mouse was placed in the start arm and allowed access to the start arm and one other arm (the familiar arm) for a 10 min session. A removable opaque door blocked access to the third arm. At the conclusion of Trial 1, the mouse was placed in a temporary holding cage for 10 min. In the memory test (Trial 2), the opaque door was removed, and the subject was returned to the start location, free to explore all three arms for 5 min. The designation of novel arm and familiar arm

is counter-balanced across animals. A camera mounted above the maze and interfaced with the Ethovision XT 12 software (Noldus Information Technology) automatically records distance traveled, arm entries, and time spent in each arm. The maze was cleaned with 50% ethanol and thoroughly dried between trials. Preference score = time spent in the novel arm/(time spent in the novel arm + time spent in the familiar arm)x100.

Morris Water Maze Test

Spatial learning and memory were assessed in the Morris Water Maze following previously described protocols (Vorhees and Williams, 2006;Yang et al., 2012). In our pilot experiments, 20 months old mice of both genotypes were able to locate the hidden platform using visual cues within 5-7 days (data not shown). For this reason, no visible trials were run before or after hidden platform trials in the current study. The 122 cm circular pool was filled 45 cm deep with tap water and rendered opaque with the addition of nontoxic white paint (Crayola). Water temperature was maintained at 23°C±1. The proximal cue was one sticker taped on the inner surface of the pool, approximately 20 cm above the water surface. Trials were videotaped and scored with Ethovision XT 12 (Noldus). Acquisition training consisted of four trials a day for 7 days. Each training trial began by lowering the mouse into the water close to the pool edge, in a quadrant that was either right of, left of, or opposite to, the target quadrant containing the platform (12 cm in diameter). The start location for each trial was alternated in a semi-random order for each mouse. The hidden platform remained in the same quadrant for all trials during acquisition training for a given mouse, but varied across subject mice. Mice were allowed a maximum of 60 s to reach the platform. A mouse that failed to reach the platform in 60 s was guided to the platform by the experimenter, and distance swam is based on visual tracking data collected within the 60s. Mice were left on the platform for approximately 15 s before being removed. After each trial, the subject was placed in a cage lined with absorbent paper towels and allowed to rest under an infrared heating lamp for 1 min. Two hours after the completion of the last training trial, the platform was removed and mice were tested in a 60 s probe trial. A second probe trial was conducted 24 hours later. Parameters recorded during training days were latency to reach the platform, total distance traveled, and swim speed. Time spent in each quadrant and number of crossings over the trained platform location and over analogous locations in the other quadrants were used to analyze probe trial performance. Proximal cue was one A4 size black and

white cartoon image taped on the inner surface of the pool, 25 cm above the surface of the water. Room (distal) cues include door, ceiling light fixture and camera, few items stored in fixed locations in the room, and a computer on a desk (not shown in the photo in Figure 2I).

Fear Conditioning

Fear conditioning was assessed following previously described protocols (Yang et al., 2012). Training and conditioning tests are conducted in two identical chambers (Med Associates, E. Fairfield, VT) that were calibrated to deliver identical foot shocks. Each chamber was 30 cm × 24 cm × 21 cm with a clear polycarbonate front wall, two stainless side walls, and a white opaque back wall. The bottom of the chamber consisted of a removable grid floor with a waste pan underneath. When placed in the chamber, the grid floor connected with a circuit board for delivery of scrambled electric shock. Each conditioning chamber was placed inside a sound-attenuating environmental chamber (Med Associates). A camera mounted on the front door of the environmental chamber recorded test sessions which were later scored automatically, using the VideoFreeze software (Med Associates, E. Fairfield, VT). For the training session, each chamber was illuminated with a white house light. An olfactory cue was added by dabbing a drop of imitation lemon flavoring solution (1:100 dilution in water) on the metal tray beneath the grid floor. The mouse is placed in the test chamber and allowed to explore freely for 2 min. A pure tone (5kHz, 80 dB) which serves as the conditioned stimulus (CS) was played for 30 s. During the last 2 s of the tone, a foot shock (0.5 mA) was delivered as the unconditioned stimulus (US). Each mouse received three CS-US pairings, separated by 90 s intervals. After the last CS-US pairing, the mouse was left in the chamber for another 120 s, during which freezing behavior is scored by the VideoFreeze software. The mouse was then returned to its home cage. Contextual conditioning is tested 24 h later in the same chamber, with the same illumination and olfactory cue present but without foot shock. Each mouse was placed in the chamber for 5 min, in the absence of CS and US, during which freezing is scored. The mouse was then returned to its home cage. Cued conditioning is conducted 48 h after training. Contextual cues were altered by covering the grid floor with a smooth white plastic sheet, inserting a piece of black plastic sheet bent to form a vaulted ceiling, using near infrared light instead of white light, and dabbing vanilla instead of lemon odor on the floor. The session consisted of a 3 min free exploration period followed by 3 min of the identical CS tone (5kHz, 80dB). Freezing was scored during

both 3 min segments. The mouse was then returned to its home cage. The chamber was thoroughly cleaned of odors between sessions.

% freezing on Day 1 was analyzed to indicate the immediate reaction to receiving foot shocks, % freezing on Day 2 and Day 3 was analyzed to reflect contextual conditioning and cued conditioning, respectively.

Open Field Test

The Open Field is the most commonly used test for spontaneous exploratory activity in a novel environment, incorporating measurements of locomotion and anxiety-like behaviors. The Open Field test was performed following previously described protocols (Yang et al., 2012). Exploration was monitored during a 30 min session with Activity Monitor Version 7 tracking software (Med Associates Inc.). Briefly, each mouse was gently placed in the center of a clear Plexiglas arena (27.31 x 27.31 x 20.32 cm, Med Associates ENV-510) lit with dim light (~5 lux), and is allowed to ambulate freely. Infrared (IR) beams embedded along the X, Y, Z axes of the arena automatically track distance moved, horizontal movement, vertical movement, stereotypies, and time spent in center zone (14.29 x 14.29cm). Data are analyzed in six, 5-min time bins. Arenas are cleaned with 70% ethanol and thoroughly dried between trials.

Immunohistochemistry

In order to confirm tau pathology was starting to arise in middle-age animals (Andorfer et al., 2003), paraffin-embedded brain sections (5 µm thick) of 12 months old mice were transcardially perfused with phosphate-buffered saline (PBS) followed by 4% paraformaldehyde (PFA; Cat# 15710, Electron Microscopy Sciences) in PBS. Brains were harvested and drop-fixed in 4% PFA in PBS at 4°C overnight, followed by incubation in 30% sucrose (Sigma-Aldrich) in PBS until the brains sank to the bottom of the container. Paraffin-embedded sections (5 µm thick) of these brains were deparaffinized in Histo-Clear II (National Diagnostics, Atlanta, GA, USA) and processed for immunohistochemistry using anti-phospho-TauSer202-Thr205 antibody (AT8; Thermo Scientific; 1:500) following previously described protocols (Andorfer et al., 2003;Santa-Maria et al., 2012) and manufacturer's protocol (MOM kit; Vector Labs, Burlingame, CA, USA, Cat # PK-2200) with some modifications. A 30-minute incubation with 3% H_2O_2/10% methanol/0.25% Triton X-100 was used to block endogenous peroxidase activity. 3,3′-

diaminobenzidine was used as a peroxidase substrate (Vector DAB Substrate Kit for Peroxidase; Vector Labs, Cat # SK-4100). Tissue sections were counterstained with hematoxylin and mounted using Cytoseal 60 (Thermo Scientific, Cat # 8310-16). The stained sections on slides were inspected and imaged by light microscopy (Olympus BX53 Microscope).

Data analysis

The statistical significance was determined using Prism (GraphPad Software). All data is presented as mean ± SEM with a p-value <0.05 considered statistically significant. "NS" indicates not significant (p>0.05).

Supplementary References

Andorfer, C., Kress, Y., Espinoza, M., De Silva, R., Tucker, K.L., Barde, Y.A., Duff, K., and Davies, P. (2003). Hyperphosphorylation and aggregation of tau in mice expressing normal human tau isoforms. *J Neurochem* 86**,** 582-590.

Santa-Maria, I., Diaz-Ruiz, C., Ksiezak-Reding, H., Chen, A., Ho, L., Wang, J., and Pasinetti, G.M. (2012). GSPE interferes with tau aggregation in vivo: implication for treating tauopathy. *Neurobiol Aging* 33**,** 2072-2081.

Sukoff Rizzo, S.J., Anderson, L.C., Green, T.L., Mcgarr, T., Wells, G., and Winter, S.S. (2018). Assessing Healthspan and Lifespan Measures in Aging Mice: Optimization of Testing Protocols, Replicability, and Rater Reliability. *Curr Protoc Mouse Biol* 8**,** e45.

Vorhees, C.V., and Williams, M.T. (2006). Morris water maze: procedures for assessing spatial and related forms of learning and memory. *Nat Protoc* 1**,** 848-858.

Yang, M., Bozdagi, O., Scattoni, M.L., Wohr, M., Roullet, F.I., Katz, A.M., Abrams, D.N., Kalikhman, D., Simon, H., Woldeyohannes, L., Zhang, J.Y., Harris, M.J., Saxena, R., Silverman, J.L., Buxbaum, J.D., and Crawley, J.N. (2012). Reduced excitatory neurotransmission and mild autism-relevant phenotypes in adolescent Shank3 null mutant mice. *J Neurosci* 32**,** 6525-6541.
